# A Mobile-Based Community Health Management Information System for Community Health Workers and Their Supervisors in 2 Districts of Zambia

**DOI:** 10.9745/GHSP-D-16-00275

**Published:** 2017-09-27

**Authors:** Godfrey Biemba, Boniface Chiluba, Kojo Yeboah-Antwi, Vichaels Silavwe, Karsten Lunze, Rodgers K Mwale, Scott Russpatrick, Davidson H Hamer

**Affiliations:** a Zambian Centre for Applied Health Research and Development Limited, Lusaka, Zambia.; b Center for Global Health and Development and Department of Global Health, Boston University School of Public Health, Boston, MA, USA.; c Ministry of Health, Child Health Unit, Lusaka, Zambia.; d Department of Medicine, Boston University School of Medicine and Boston Medical Center, Boston, MA, USA.; e United Nations Children's Fund (UNICEF), Lusaka, Zambia.; f Akros, Lusaka, Zambia.; g Section of Infectious Diseases, Department of Medicine, Boston University School of Medicine, Boston, MA, USA.

## Abstract

**Introduction:**

Effective community health management information systems (C-HMIS) are important in low-resource countries that rely heavily on community-based health care providers. Zambia currently lacks a functioning C-HMIS to provide real-time, community-based health information from community health workers (CHWs) to health center staff and higher levels of the health system.

**Program Description:**

We developed a C-HMIS mobile platform for use by CHWs providing integrated community case management (iCCM) services and their supervisors to address challenges of frequent stock-outs and inadequate supportive supervision of iCCM-trained CHWs. The platform used simple feature mobile phones on which were loaded the District Health Information System version 2 (DHIS2) software and Java 2 platform micro edition (J2ME) aggregation and tracker applications. This project was implemented in Chipata and Chadiza districts, which supported previous mHealth programs and had cellular coverage from all 3 major network carriers in Zambia. A total of 40 CHWs and 20 CHW supervisors received mobile phones with data bundles and training in the mobile application, after which they implemented the program over a period of 5.5 months, from February to mid-July 2016. CHWs used the mobile phones to submit data on iCCM cases seen, managed, and referred, as well as iCCM medical and diagnostic supplies received and dispensed. Using their mobile phones, the supervisors tracked CHWs' reported cases with medicine consumption, sent CHWs feedback on their referrals, and received SMS reminders to set up mentorship sessions.

**Observations:**

CHWs were able to use the mobile application to send weekly reports to health center supervisors on disease caseloads and medical commodities consumed, to make drug and supply requisitions, and to send pre-referral notices to health centers. Health center staff used the mobile system to provide feedback to CHWs on the case outcomes of referred patients and to receive automated monthly SMS reminders to invite CHWs to the facility for mentorship. District- and central-level staff were able to access community-level health data in real time using passwords.

**Lessons Learned:**

C-HMIS, using simple feature phones, was feasible and viable for the provision of real-time community-based health information to all levels of the health care system in Zambia, but smartphones, laptops, or desktop computers are needed to perform data analysis and visualization. Ongoing technical support is needed to address the hardware and software challenges CHWs face in their day-to-day interaction with the application on their mobile phones.

## Abstract (precis)

Using simple-feature mobile phones, CHWs sent weekly reports on disease caseloads and commodities consumed, ordered drugs and supplies, and sent pre-referral notices to health centers. Supervisors provided feedback to CHWs on referred patient outcomes and received monthly SMS reminders to set up mentoring sessions with the CHWs. Scale-up limitations include: (1) staff shortages at health centers to supervise the CHWs, (2) need for ongoing technical support to troubleshoot challenges with mobile phones and software, and (3) recurring costs for data bundles.

## INTRODUCTION

According to Theo Lippeveld, at the time he was working for the Rebuilding Basic Health Services project in Monrovia, Liberia, “only what gets measured gets attention.”^1^ But it is also true that only what gets collected gets measured, and only what gets recorded or documented and disseminated gets the attention of policy makers and service providers.

Lippeveld argued that the availability of information on health services performance can empower community health workers (CHWs) and their supervisors to improve the quality of community-based health services. He added, “there is [also] a need to link the health information generated by CHWs to the facility-based routine health information systems …. Yet, in most countries, this vital information on health services provided by CHWs is not routinely captured.”^1^ These observations are very much true for Zambia. The importance of a robust, well-functioning community health management information system (C-HMIS) cannot be overemphasized.

Globally, mHealth technologies are increasingly used by health systems to improve health worker effectiveness by documenting relevant data.^2^^–^^4^ To that end, the Zambian government set the goal to “improve hospital and community level health information, by ensuring their access to fully functional HMIS” in its *National Health Strategic Plan 2011–2015*.^5^ In Zambia, the current national HMIS captures data only from the health-facility level and up, including some data from health posts reported by a new cadre of staff called community health assistants (CHAs). The system, however, does not capture data collected by community-based agents like CHWs. Although a paper-based reporting system that transmits data from CHWs to health facilities does exist, it is not fully functional; this is due in part to the challenges of sustaining availability of reporting forms and delivering completed forms to the health centers.

The Zambian Centre for Applied Health Research and Development (ZCAHRD) and Boston University, in collaboration with the United Nations Children's Fund (UNICEF), Ministry of Health (MOH), and Akros, are implementing an mHealth-enhanced C-HMIS. The C-HMIS is an adaptation of District Health Information System version 2 (DHIS2) software, referred to as community DHIS2 (C-DHIS2) mobile in Eastern Province, Zambia.

The C-DHIS2 was implemented in the context of a cluster randomized controlled trial (RCT) that aimed to improve the quality of integrated community case management (iCCM) of malaria, pneumonia, and diarrhea (Clinicaltrials.gov protocol ID# 4980/A0/04/001/010). This article describes the technical feasibility and implementation of this innovative C-DHIS2 and the rural setting in which the mHealth innovation was implemented; full results of the clinical trial will be reported elsewhere. The aim of the project was to provide information on implementation issues related to C-DHIS2 to the Zambian government and to countries that have no C-HMIS or where paper-based community-to-facility reporting is faced with logistical challenges. The pilot project was implemented in the natural Zambian health system context, with the introduction of mHealth as the only intervention.

## ZAMBIAN HEALTH SYSTEM CONTEXT

The Zambian health service delivery system operates at 3 levels:

1. Primary health care services serve as the first point of contact and are located within communities and districts. Within this category are district hospitals, health centers, health posts, and community-based health care services, which provide a range of promotive, preventive, curative, and rehabilitative services. Health posts are staffed by nurses, clinical officers, environmental health technologists, or CHAs. CHAs are a fairly new cadre of paid health workers who are trained for 12 months and deployed to the health posts to work hand in hand with the CHWs. However, because their numbers are small, they have not yet been fully integrated into the community health system.
2. Facilities at the provincial level serve as referral points from the primary level.
3. Teaching and specialized referral hospitals comprise the third level.

Although CHWs are not part of the formal health care system—as they are volunteers and not paid by the government—they are recognized and trained by government or its partners using a government-approved curriculum. CHWs are members of the communities where they work, and are selected by and answerable to their communities.^6^ Official government literature in Zambia describes CHWs as “members of communities who work either for pay or as volunteers in association with the local health care system … and usually share ethnicity, language, socioeconomic status and life experiences with the community members they serve.^7^ In Zambia, these CHWs bear different titles—from health promoters, community health advisors, lay health advocates, lay counselors, and community health representatives to peer health educators and malaria agents—depending on their day-to-day duties. Some CHWs have been specifically trained in iCCM, a program being rolled out and scaled up countrywide in Zambia. These CHWs were trained in the diagnosis and management of malaria, pneumonia, and diarrhea—based on a World Health Organization (WHO)-recommended iCCM training package—and use of the WHO *Sick Child Recording Form* job aid. In general, CHWs provide preventive, promotive, and basic curative services to people of all ages at the community level.

## PROJECT CONTEXT

The implementation of the C-DHIS2, described in this article, is part of a cluster RCT implemented in Chipata and Chadiza districts of the Eastern Province of Zambia. A cluster was defined as a health center/post, and included all CHWs in its catchment area that were providing iCCM and all children under 5 years old who presented with suspected malaria, pneumonia, or diarrhea to the CHWs. Intervention clusters included CHWs trained in both mHealth and the basic 7-day MOH iCCM training, while control clusters included CHWs who had received only the basic 7-day MOH iCCM training. The primary outcome for the cluster RCT was the proportion of children under 5 appropriately treated for malaria, diarrhea, or pneumonia by CHWs. The intervention used mHealth-enhanced inventory management and supportive supervision and mentorship. A total of 80 CHWs were trained under the cluster RCT, 60 from Chipata (30 intervention, 30 control) and 20 from Chadiza (10 intervention, 10 control).

Chadiza district has 14 health facilities—including 4 health posts, 6 rural health centers, 2 zonal community health centers, 1 urban community health center, and 1 level-1 (district) hospital—that serve a population of about 92,300. In contrast, Chipata district has 42 health facilities—including 7 health posts, 30 rural health centers, 4 urban health centers, and 1 level-2 (provincial) hospital—that serve an estimated population of 486,500.

## THE DHIS2 mHEALTH PLATFORM

The Zambian national MOH HMIS is built on the DHIS2 database platform. The DHIS2 is the leading global open-source data warehousing software for low-resource countries. It combines multiple modalities of data capture, aggregated form, entity tracking, and single event data, with streamlined data warehousing and data analytics tools. The DHIS2 also serves as the national database platform for the Ministry of Local Government and Housing (MLGH) and is being piloted by the Ministry of General Education (MOGE). The widespread use of the platform has built a strong foundation of DHIS2 technical expertise in Zambia. To that end, we chose the DHIS2 mobile platform because: (1) it can be easily integrated and adapted into the existing MOH's HMIS, (2) the MOH also already possesses the technical knowledge to integrate and maintain the C-DHIS2, and (3) the DHIS2 works well with data entry Java applications, particularly DHIS2 mobile.

In order to run the DHIS2 mobile application, a mobile feature phone must be able to support Java, support installation of Java-based applications, and have a heap size of at least 512 KB. The heap size describes how much of the phone's memory an application can use.

While it is easy to find out if a phone supports Java applications—this information can usually be found in the phone's user manual or online—it is more difficult to determine the phone's heap size, since this is not a specification that is normally of interest to an everyday user. Phone manufacturers rarely supply the information, and service personnel do not usually have this information. However, there are 3 ways of finding a phone that supports DHIS2:

1. Purchase a device that is already known to be able to run the application
2. Buy a phone in a high enough price range so it is more likely to have the required specifications
3. Acquire a selection of cheap non-carrier-provided phones and test each to see if they will run the application

We selected the third option, deciding not to use carrier-provided phones, and visited a phone shop with a good selection of models. Several models seemed to fit the required specifications, but the most promising one was the Samsung B313E, which is widely available in local markets across the country. This phone was tested and found to be suitable for use with DHIS2 mobile. The phone has a 2-inch screen and a basic 3x4 number pad with T9 predictive text technology (Figure). The project procured these phones for CHWs and provided data bundles through a selected mobile network service provider.

**FIGURE:**
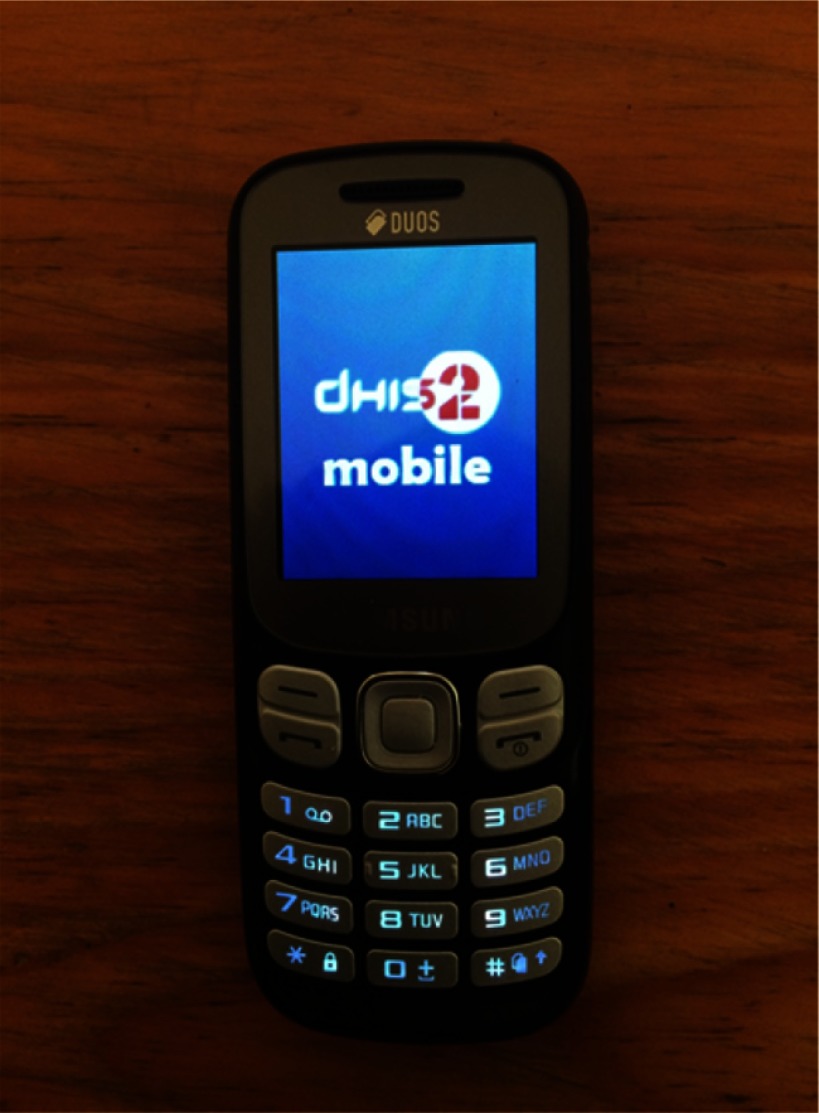
Samsung B313E Mobile Phone Used by Community Health Workers in the Community Health Management Information System Program

The benefit of having a non-carrier branded phone is that it will work with SIM cards from any telecommunication provider because it is not locked to a single carrier. If the program is scaled nationwide, unlocked phones will allow the implementing team to choose the network with the best coverage in a given area.

The C-DHIS2 mobile consists of 2 separate applications: the DHIS2 Java 2 platform micro edition (J2ME) aggregate capture application and patient tracker application. Both applications are enabled for offline data entry if a mobile network connection is not available, and will automatically send the data once the phone reconnects with a mobile network. The following is a summary of the functionalities of the 2 applications:

**Aggregate Application:** This application is used to capture the number of cases seen, managed, and referred by CHWs, and reports and requisitions for iCCM drugs—artemether-lumefantrine, amoxicillin, oral rehydration solution (ORS), and zinc—and diagnostic tools, such as malaria rapid diagnostic tests (RDTs), on a weekly basis. Three forms may be selected for data entry by the CHWs:

- The *iCCM Disease Management Form*, which is used to report the number of cases seen, cases referred, and deaths from diseases such as diarrhea (bloody and non-bloody), pneumonia, and RDT-confirmed malaria.
- The *Reports and Requisition Form*, which is used to report stock information on artemether-lumefantrine, amoxicillin, zinc, ORS sachets, and RDTs.
- The *Newborn Care at Home Form*, which captures the number of newborn babies who have been visited or referred or have died in the CHW catchment area.

**Patient Referral Tracker and Alert Application:** This application is used to track patients who have been referred by CHWs to health facilities and to provide feedback from health facility staff to CHWs on the referred patients. The tracker application also supports mentorship activities and provides alerts for the submission of mentorship reports. This consists of 2 major components:

- The patient referral tracker is used to notify clinicians/health center staff of the name of the patient referred to the clinic, the observed signs and symptoms, and any pre-referral treatment administered. Pre-referral messages are only sent to a specific mHealth-enabled mobile phone at the health facility. To ensure that the messages reach the clinician on duty, the receiving mobile phone is usually left with a specific person on duty. The referral tracker is also used by the clinicians to provide feedback to CHWs on the case outcome of referred patients—referred to hospital, treated and discharged, or deceased.
- The mentorship report alerts the supervisor when it is time to schedule a mentoring session. Once a month, an automated short message service (SMS) message is sent out to the supervisors to remind them to conduct mentorship with the CHWs under their supervision. After the mentorship session at the health facility, the supervisor records the 3 main weaknesses observed, enters them on the mentorship report through the simple feature phone, and submits the results to the C-HMIS after providing feedback to the CHW. While the recording of weaknesses may appear negative, it is meant to identify issues that require supervisor intervention and follow-up support. During the mentorship sessions, however, the supervisor does make sure to recognize and appreciate a CHW's good performance.

An important feature of this mHealth innovation is the online availability of the application to supervisors at facility, district, provincial, and national levels. This makes the iCCM data visible at all levels, making it a potential management tool for analyzing community-based health management information and for decision making.

## PROGRAM DESCRIPTION

Within this program, the C-DHIS2 was implemented within the context of iCCM. Zambia adopted the iCCM strategy in May 2010 and has since been scaling up the program countrywide with support from donors such as UNICEF and the Global Fund to Fight AIDS, Tuberculosis and Malaria (the Global Fund). Although the CHWs in our study sites were the first to be trained in iCCM in these 2 districts, the training used the national curriculum and national trainers that were already part of the national iCCM training scale up. As of December 31, 2015, 4,002 CHWs and 833 health center supervisors had been trained in 58 of 106 districts.^1^ In addition to scaling up training, the country was also scaling up iCCM implementation nationwide. Trained CHWs used the *Sick Child Recording Form* as a job aid to diagnose, treat, and report on management of malaria, diarrhea, and pneumonia for children under 5. However, 2 major challenges to the effective implementation of iCCM in Zambia were identified: frequent stock-outs and inadequate supportive supervision of iCCM-trained CHWs.^8^^–^^10^

To address these challenges, ZCAHRD designed a pilot implementation science research study entitled, ”Strengthening the delivery of integrated community case management (iCCM) in two districts of Eastern Province, Zambia.“ The aim of the project was to strengthen the delivery of iCCM in Chipata and Chadiza districts of the province supported through mHealth technology, improved supply chain management of iCCM commodities, and enhanced supportive supervision of iCCM-trained CHWs. In collaboration with Akros, ZCAHRD designed and developed the iCCM C-DHIS2 mobile application, which was adapted from the existing DHIS2 platform. As part of the program, 80 CHWs and 40 CHW supervisors/health facility staff were trained in a basic 7-day iCCM training course using a standard MOH training curriculum.^11^ The health facility supervisors were either nurses or clinical officers. In addition to the training provided by the project, all the CHWs, both intervention and control, were provided with job aids, in form of the *Sick Child Recording* Form; acute respiratory infection timers; a bicycle; gum boots; and a bag. The CHWs (n=40) and their supervisors (n=20) in the intervention arm of the pilot project were also given a feature mobile phone, loaded with DHIS2 mobile. While the phones could have been used for conversations and activities beyond iCCM duties, a snap survey undertaken as part of the costing exercise showed that the phones were rarely used outside of iCCM work. Although the phones could have been used to send or receive voice calls, the CHWs were provided with data bundles only, meaning any voice calls would not be covered by the project. All the CHW supervisors were trained in iCCM supervisory skills. Following their training in iCCM, both CHWs and their supervisors in the intervention arm were trained in the C-DHIS2 mobile application. After training, all the CHWs and their supervisors implemented the program over a period of 5.5 months, from February 2016 to mid-July 2016.

Below is a summary description of the mHealth intervention activities by role.

**CHWs:** On a weekly basis, CHWs summarized iCCM cases seen, managed, and referred, used the data to complete the electronic forms on the applications installed on their mobile phones, and submitted those forms electronically to the DHIS2 database. They also summarized iCCM medicine and diagnostic tools received and dispensed, determined the order quantity using allowable maximum stock levels, and then completed an electronic Reports and Requisition form installed on their mobile phones and submitted it electronically to the DHIS2 database. For each sick child with danger signs, CHWs completed and submitted an electronic referral note with a unique patient ID, personal details, signs and symptoms, diagnosis, and pre-referral treatment, if any.

**Supervisors:** On a regular basis, the CHW supervisors logged into C-DHIS2 and accessed weekly reports submitted by their CHWs, and compared cases reported with medicine consumption, such as the number of positive RDTs compared with the number of artemether-lumefantrine tablets dispensed. The supervisors identified who was reporting and followed up those who did not in order to determine why they were not reporting. The supervisors received electronic referral notes on their mobile phones and sent electronic feedback notes to their CHWs on their mobile phones. On a monthly basis, supervisors received mentorship SMS reminders and invited their CHWs to their health centers and provided mentorship. After each mentorship session, the supervisors completed a mentorship report and submitted it electronically to the DHIS2 database. Based on the Reports and Requisition forms received from CHWs, supervisors supplied medicines and RDTs to CHWs, ordering the CHWs' drugs and RDTs alongside their own medicines and diagnostic tools from the district pharmacist.

## OBSERVATIONS FROM EARLY FIELD IMPLEMENTATION

From early in the implementation process, we had 3 key observations. The first was that the automation of the patient referral, feedback, and SMS reminders for monthly mentorship were unique features of this pilot, which was the first of its kind. The weakness in most community referral systems has been the provision of feedback to CHWs.^12^^–^^14^ This particular iCCM C-DHIS2 platform provided a solution to the feedback problem. Although we did not carry out an evaluation of the effect of this feedback on CHWs, the results from a study from Uganda suggest that such feedback could be highly motivating because communication between health workers and supervisors had increased.^15^ The second observation was that because an electronic referral note—with all patient details, including diagnosis made and pre-referral treatment, if any—reached the health center before the patient arrived, it gave health center staff the opportunity to prepare for the arrival of the patient. It also enabled health center staff to follow up on referred patients that did not reach the health facility. Our final observation was that the automated monthly SMS reminders sent to health center staff to invite CHWs to the facility for mentorship were enhancements to current supervisory activities—routine quarterly supportive supervision that combines iCCM and all other health-related supervision of CHWs. Considering the low staffing levels at most rural health facilities and the amount of work done at the facility, including travel out of stations for workshops, using the C-DHIS2 as a monitoring and supervisory tool is expected to improve technical efficiencies in the implementation of iCCM in contexts similar to the areas where this project is being implemented.

## LESSONS LEARNED

The simple feature phones were able to accommodate the reporting applications and were suitable for uploading reports into the DHIS2 database. However, they were not useful for the visualization or analysis of data. Therefore, health center-, district-, and central-level supervisors and users of C-DHIS2 data need to have smartphones, laptops, or desktop computers to perform data analysis. We also observed that routine technical support—troubleshooting and fixing both software-related problems (related to the applications) and hardware-related problems (related to the phones and SIM cards) —was required to address the technical challenges CHWs face in their day-to-day interaction with the application on their mobile phones. While mobile network and Internet connectivity limited the use of the applications in selected parts of the district, no problems were encountered in the submission of weekly reports. This was likely due to the ease of opening the applications after the CHWs successfully logged in the first time: they did not have to log on the next time they needed to use the application, except when the application had to be initialized or the CHW accidentally clicked on the initialize option. In a few isolated locations with mobile network connectivity challenges, weekly report forms were completed offline and then sent once the CHW reached a location where the network is available. The biggest challenge to using this program was sending and receiving patient referrals in those locations with limited network access. For the referral form to be sent and received, the CHW's mobile phone and the receiving health facility mobile phone had to be in locations where the network was available. In cases where connectivity was an issue, the reporting or referral forms were completed offline and as soon as network access was available, the reports were sent from the CHW's mobile phone and received by the health facility supervisor.

### Sustainability and Cost Implications

Each mobile phone costs the equivalent of US$38.50. On a monthly basis, the selected network service providers send data bundles worth US$5.00 to all CHWs' and supervisors' mobile phones. To scale up this program, recurring costs would have to be budgeted for in the government district health plan. The government of Zambia has already requested an estimate of the full cost of implementing this program throughout the country, including set-up and recurrent costs. A costing exercise has been completed and the report is almost finalized for submission to the donor; we will publish the results in a separate paper. The cost information will be shared with the Zambian government to inform its scale-up planning. The project was designed in such a way that it can be sustained after the project funding ends, by making sure that it is fully owned by the Zambian MOH at all levels. MOH officials at the central and district levels are part of the implementation team, and plans are underway to hand over the program to the government once the implementation research study ends. iCCM, as a strategy, is currently being scaled up countrywide with support from the government of Zambia and partners, such as UNICEF and the Global Fund. Since the MOH has decided to integrate the C-DHIS2 into the national DHIS2 HMIS, its implementation and scale up after the pilot will be funded as part of the national HMIS. The MOH has plans to engage network service providers to highly subsidize the cost of data bundles and consider the arrangement as part of the company's corporate social responsibility.

### Limitations for Delivery to Scale and Data Use

The main limitation to scale up of this intervention is continued shortage of staff at the health-center level, particularly the nurses and clinical officers who would supervise the CHWs. This problem may be resolved by the addition of the new cadre of CHAs. As of September 2016, 1,403 CHAs had been trained and deployed across 597 health posts throughout Zambia, serving around 4 million people. The country's 2 CHA training schools currently have the potential to graduate at least 500 CHAs annually.^16^ These CHAs can be trained in iCCM and the use of mHealth technology in order to increase the number of supervisors at the facility level.

Another limitation is the need for ongoing technical support to troubleshoot technical challenges with the mobile phones. This issue can be overcome by empowering district iCCM coordinators and the district information officers with the skills to troubleshoot the functionalities of the mobile DHIS2 application. Although financial sustainability might be a limitation in the future, the current government's commitment to integrating this application into the national DHIS2 means that the national budget should cover costs and might mobilize support from mobile service providers to provide free or highly subsidized data bundles. While data utilization may be a challenge—in the light of shortage of staff at the health center level—more sensitization of providers, structured data review forums, and the creation of the DHIS2 dashboards may motivate the staff at the health center, district, and higher levels of the health system to use the data generated for decision making.

### Contextual Adaptability and Replicability

This application has only been implemented as part of a pilot study in 2 districts of Zambia. However, since it uses the basic DHIS2 platform and stock DHIS2 Java applications, which are currently being implemented nationwide, we do not see a challenge in adapting and/or replicating the implementation elsewhere in Zambia or in other countries where DHIS2 is also used as an mHealth platform.

### Data Security

Access to the mHealth system can be gained by users and super users—those with special privileges—through user name and password-protected authorization.

### Compliance with National Guidelines

This pilot is in compliance with the Information and Communications Technology Act No. 15 of 2009 and the 2006 National Information and Communications Policy of Zambia.^17^^,^^18^ Additionally, the development and implementation of the C-HMIS tool is in agreement with the MOH's e-Health Strategy 2014–2016, which expresses the objective, “to increase access to quality of healthcare and health-related information through the use of mobile technologies.”^19^

### Fidelity of the Intervention

The intervention delivered what it was programmed to deliver, and proved to be reliable and robust enough to accommodate indicators from other community health programs. As a result, a roadmap has been designed to transform this intervention into a national C-HMIS. However, the technical sustainability of this intervention depends on (1) the provision of regular technical support to users, especially CHWs; and (2) the provision of data bundles. The latter would ideally be accomplished by contracting with network service providers to provide data bundles to all registered SIM cards consistently, weekly or monthly, over an agreed period of time—through reverse billing.

## Notes

**Peer Reviewed**

**First Published Online:** 2017 August 30
